# The ethical landscape of professional care in everyday practice as perceived by staff: A qualitative content analysis of ethical diaries written by staff in child and adolescent psychiatric in-patient care

**DOI:** 10.1186/1753-2000-6-18

**Published:** 2012-07-09

**Authors:** Veikko Pelto-Piri, Karin Engström, Ingemar Engström

**Affiliations:** 1 Psychiatric Research Centre, Örebro County Council, Box 1613, SE-701 16, Örebro, Sweden; 2 School of Health and Medical Sciences, Örebro University, SE-701 82, Örebro, Sweden; 3 School of Humanities, Education, and Social Sciences, Örebro University, SE-701 82, Örebro, Sweden

**Keywords:** Staff, Child and adolescent psychiatric care, Ethical considerations, Diary method, Qualitative content analysis, Ethical issues

## Abstract

**Background:**

Although there has been some empirical research on ethics concerning the attitudes and approaches of staff in relation to adult patients, there is very little to be found on child and adolescent psychiatric care. In most cases researchers have defined which issues are important, for instance, coercive care. The aim of this study was to provide a qualitative description of situations and experiences that gave rise to ethical problems and considerations as reported by staff members on child and adolescent psychiatric wards, although they were not provided with a definition of the concept.

**Methods:**

The study took place in six child and adolescent psychiatric wards in Sweden. All staff members involved with patients on these wards were invited to participate. The staff members were asked to keep an ethical diary over the course of one week, and data collection comprised the diaries handed in by 68 persons. Qualitative content analysis was used in order to analyse the diaries.

**Results:**

In the analysis three themes emerged; 1) good care 2) loyalty and 3) powerlessness. The theme ‘good care’ contains statements about the ideal of commitment but also about problems living up to the ideal. Staff members emphasized the importance of involving patients and parents in the care, but also of the need for professional distance. Participants seldom perceived decisions about coercive measures as problematic, in contrast to those about pressure and restrictions, especially in the case of patients admitted for voluntary care. The theme ‘loyalty’ contains statements in which staff members perceived contradictory expectations from different interested parties, mainly parents but also their supervisor, doctors, colleagues and the social services. The theme ‘powerlessness’ contains statements about situations that create frustration*,* in which freedom of action is perceived as limited and can concern inadequacy in relation to patients and violations in the workplace.

**Conclusions:**

The ethical considerations described by child and adolescent psychiatric care staff are multifaceted and remarkably often concern problems of loyalty and organization. These problems frequently had a considerable influence on the care provided. It seems that staff members lack a language of ethics and require both an ethical education and a forum for discussion of ethical issues.

## Background

A host of ethical problems inherent in psychiatric in-patient care have to be considered by staff members, especially in relation to coercive care. Moreover*,* ethical problems specific to child and adolescent psychiatric care are not found in the adult equivalent [1,2]. It is necessary to consider the age and degree of maturity of the young person both from a legal and a psychological point of view. More attention has recently been focused on the rights of the child within medical care, especially in view of the Convention on the Rights of the Child [3]. In Sweden*,* this has among other things led to children having the right of secrecy in relation to their parents from the age of 15 [4]. At the same time as the rights of the young patient must be respected, the parents need both information and support to be able to assume their parental responsibility, something that psychiatric care has to balance in an ethically reasonable way [1]. There are also more stakeholders in relation to child and adolescent psychiatric care compared to the adult equivalent, such as schools and social services, something that creates more interfaces where conflicts of interest may emerge, since the primary concern of child and adolescent psychiatric care is the protection of the rights of the child [2]. According to Swedish legislation*,* this protection should be characterized by the young person participating in the care and decisions concerning its content. In an ideal situation, this participation can give the young person an understanding of the meaning of care [5].

The greatest focus on staff members’ own perceptions about what they consider ethical problems or issues is found in somatic medical care research. Some of the problems or issues reported by nurses in geriatric care, acute medical care and school health were 1) that they often did not agree with the doctor on care measures [6,7], 2), that demands from the organization, parents or relatives were not considered beneficial to the patient [6,8], 3)*,* that routine-centred care left little or no place to take account of the needs and wishes expressed by patients [6] and 4) that a strong group identity within the team where loyalty was expected was an obstacle to reporting incidents of neglect or abuse by colleagues [6].

Corresponding studies within adult psychiatric care have mostly focused on ethical issues defined by the researcher and how these were perceived by care staff in relation to coercive care, coercive measures and moral stress. We have not found any corresponding empirical studies in the field of child and adolescent psychiatric care. Nurses in adult psychiatric care often have a heavy work load; they have to administer coercive measures and often respond to violence from patients. Some studies indicate that coercive measures nevertheless are rarely considered ethically problematic by nurses [9,10]. The heavy workload, however, creates emotional conflicts. To manage these conflicts, nurses adopted a professional role; diagnosing the patients’ behaviour, avoiding ordinary everyday conversations with them and adhering to formal and informal rules [11]. Furthermore, loyalty within the team was strong and prevented independent decisions. It was important for the well-being of the nurses to find a solution to the ethical problems and when that was not possible they expressed feelings of guilt, frustration and powerlessness [12]. Within nursing care research it has been claimed that moral stress arises because nurses are morally sensitive to the vulnerability of the patient, are aware of external factors that prevent them from meeting the patient’s needs and feel that they are not in control of the situation [12]. A study revealed that 52% of nurses and social workers were frustrated by the fact that they could not find solutions to their ethical problems [13].

While there is some empirical research on ethics concerning the attitudes and approaches of staff in relation to adult patients, it is rare in child and adolescent psychiatric care. In most cases, researchers have defined important issues, for instance coercive measures. In the present study, staff members described their view of the ethical landscape of professional care in everyday practice. The aim of this study was to provide a qualitative description of situations and experiences that gave rise to ethical problems and considerations as reported by staff members on child and adolescent psychiatric wards, although they were not provided with a definition of the concept.

## Methods

### Setting and participants

The present study was carried out at six child and adolescent psychiatric wards in central Sweden, providing both voluntary and coercive care. The clinics admitted children and adolescents under 18 years in need of psychiatric in-patient care from the surrounding catchment area. The patients were almost exclusively adolescents and the length of stay was usually around thirty days. The adolescents’ problems varied considerably, but the most frequent diagnoses were eating disorders, psychoses, depression with or without suicide attempts and neuropsychiatric disorders.

Staff members on the wards who worked directly with the patients were invited to participate, regardless of occupational status. Approximately half of them were mental health care assistants. Of the other half, the majority comprised registered nurses along with several doctors, psychologists, social workers and teachers. Almost all staff members had some form of health professional education. The decision to use anonymous diaries unfortunately precluded other relevant background data. Approximately 20-30 persons were employed on each ward. The number of people who handed in their diaries was 68. The response frequency varied greatly between wards, ranging from 3 to 18 persons.

### Design and procedure

The staff members were asked to keep an ethical diary over the course of one week. The diary had eight pages, the first containing instructions and the following were blank, except for the name of the day. This method was selected in order to obtain statements freely provided by staff members, thus facilitating the discovery of experiences of the phenomenon in question that would otherwise have remained concealed if employing more structured methods.

In the brief instructions, the participants were asked to describe work situations and experiences that they considered to give rise to some form of *ethical consideration*. We also enquired about what thoughts these situations inspired in them. No specific description was required, but we suggested that they should write down their experiences on a daily basis after finishing their day’s work. The instructions contained no definition of either ‘ethics’ or ‘considerations’ as the aim was to make the participants feel free in relation to these concepts. The actual formulations used in the instructions can be found in an Additional file 1.

### Analysis and interpretation

In order to analyse the diaries we used qualitative content analysis [14,15], a method considered adequate for providing a qualitative description [16]. Every statement from the 256 days that contained an ethical consideration or problem pertaining to psychiatric care or treatment was included in the analysis, which was conducted by three persons: VP, a social worker, KE, a pedagogue and IE, a child and adolescent psychiatrist. Our pre-understanding was derived from our review of the literature and our professional education and experience.

The first step of the analysis was to read through the statements several times in order to obtain an impression of the material as a whole. During the readings, each statement was considered a meaning unit [17] in which we sought considerations or problems to be summarized into a condensed meaning unit. We then interpreted the underlying meaning(s) in each statement, see Table 1. Thereafter*,* sub*-*themes and themes were created, the number of which was gradually reduced. When the interpretation of the whole material was completed, themes and sub-themes were chosen that provided a basis for a thick description [18] that best described the material as a whole [19]. The analysis involved a back and forth movement between the whole and parts of the text. In order to verify our interpretation*,* the material was rethematized on the basis of the sub*-*themes that had emerged and some adjustments were made to the final result.

**Table 1:** **An example of the thematization of a statement**[17]

Part of a meaning unit (or statement)	Condensed meaning unit	Interpretation	Sub-theme	Theme
A 12-year-old boy comes when his mother is admitted to adult psychiatric care. The police arrested the boy for shoplifting food. It appeared that the family left the refugee camp after the expulsion decision. We became a "terminus" for societal responsibility without being aware of the reason. Our opinion is irrelevant.	We must take care of a refugee boy who shoplifted without questioning the decision*.* We became a "terminus".	Powerlessness - inadequate health care – storage	A terminus for the patient	Powerlessness

The formulation and interpretation of condensed meaning units were conducted independently by VP and KE. When formulating the themes, the material was split into two parts, one of which was dealt with by VP and the other by KE. After the coding and formulation of themes, they examined each other’s material and agreed, with the help of IE, on which sub-themes and themes were the most suitable.

We aimed to strive for ecological validity [20], which means that staff working within psychiatric care should be able to recognize their own situation in our results. In the course of our research, the results of the analysis were communicated to and discussed with staff from the participating clinics, as well as with staff from other clinics. These discussions influenced the analysis, as they revealed which statements and interpretations were perceived as reasonable and could be said to reflect the experiences of the staff.

## Results

Nine sub-themes and three themes emerged in the interpretation. The themes were; 1) good care*,* 2) loyalty and 3) powerlessness, see Table 2, where the number of statements in each sub-theme is also presented.

**Table 2:** The statements in the sub-themes that emerged in the interpretation of the data

**Themes**	**Sub*-*themes**	**Number of statements**
Good care	An ideal of commitment	50
	Building alliances for good care	23
	The borders of coercion	24
	A professional distance to patients	25
Loyalty	The patient comes first	22
	Belonging to the team	14
Powerlessness	Inadequacy in relation to patients	24
	A terminus for the patient	10
	Feelings of helplessness	47
Not thematizied		17

### Good care

Participants expressed *an ideal of commitment* and the will to act professionally. They wished to provide the best possible care for patients, dare to make priorities, have good communication with parents and involve them as a resource in relation to their children. They criticized colleagues who did not show commitment, were not objectively professional or expressed unacceptable values. The team should remain calm and show respect in meetings with angry, resentful or critical parents. Participants found it problematic that there were so many different views within child and adolescent psychiatric care. Commitment to work led to confirmation from both patients and their parents, which endowed the work with meaning. There were also statements about the problem of being unable to live up to one’s own ethical ideals.

"My ethical conflict today concerns the role I ought to play as “the good nurse”. Our trade union strike, which resulted in no backup staff, doctors under stress, damned kids and the feeling of always being inadequate makes me want to stamp on the floor or just go home… but instead I remain friendly and clench my teeth… and repress my feelings, which is not a good thing. Good ethics?"

Some statements revealed that rules were broken in order to care for and meet the needs of a particular patient, for example by giving him/her hot cocoa and fruit from the closed staff room. The participants tried to find ways of showing patients respect in situations of ongoing observation or when coercive measures were employed.

The participants endeavoured to find a strategy for collaboration and *building alliances for good care* with patients and their parents. They were anxious to encourage parental involvement and tried to be flexible in order to create trust between themselves, the patient and his/her parents. Sometimes parents had their own difficulties and were unable to respond to the invitation, thus the team became very frustrated due to failure to create an alliance.

The participants seldom perceived that prescribed coercive care or measures were ethically problematic. Instead, ethical considerations were present at *the borders of coercion*, particularly in relation to pressure and restrictions applied towards patients, especially those admitted voluntarily. Staff and parents can sometimes restrict voluntary patients’ liberty as much as those in coercive care, but without the legal right to do so. Some coercive measures were also perceived as emotionally hard but ethically non-problematic.

"Today I tube-fed a patient with anorexia. She exhibited acute anguish, wept and cried when given the gruel. However, I did not experience an ethical dilemma when forcing her. If she doesn’t eat she will die, so the choice was simple."

Several statements indicated an opinion among staff about the need for *a professional distance to patients*. Criticism was directed against staff members who were too involved with patients and became like a “self-appointed extra mum”. Some participants were more personal in their approach and critical of the idea of professional distance.

### Loyalty

"I experienced an ethical dilemma during a home visit. We went there in order to provide support and advice to the son in the family who had been diagnosed with autism. The mother cried and told us that she could not make ends meet. Her debts amounted to 4,500 SEK. She said that she just could not go on any longer. We, the staff, told her that we were unable to help her and were there for her son. We suggested that she contact our social worker who might be able to help her."

Some parents have serious financial, social or psychological problems of their own. However, in such cases the needs of *the patient comes first*. It can also happen that a team member chooses to prioritize patients instead of acting in accordance with organizational directives. There are also occasions when team members believe they must be loyal to their organization and team without necessarily giving lower priority to patients or parents, as when parents criticize colleagues.

*Belonging to the team* was important and team members were expected to be loyal. The team members take care decisions on a continuous basis, thus many decisions were made when only a part of the team was present. Despite this, participants were nearly always loyal to decisions made by other team members, although they considered them to be wrong. This loyalty was described as necessary and positive because they believed that the team had to be united to avoid ‘splitting’ on the ward. Some statements revealed that care staff dared to contradict the views of their manager or the doctor, but not those of their colleagues.

"One young person for whom I was the contact person behaved badly. The following day I had the feeling that the rest of the staff wanted this young person to be removed from the ward. During a subsequent conversation with the youth, he did not seem to understand what he was being accused of and even appeared sad, thus I found it difficult to remain firm. But it was either that or giving in and arguing with the rest of the staff. It is always difficult being firm with someone who is already in an inferior position."

### Powerlessness

The participants experienced *inadequacy in relation to patients* and many statements described the ineffectiveness of the organization. In one of the wards there was a conflict that created anxiety among the staff, thereby complicating coordination. On another ward the team devoted an inordinate amount of time to planning and discussions, despite which the planning was inefficient, leading to delays and irritation among staff and patients. At many meetings with families, more than one member of the team was expected to be present and it was difficult to find a suitable time for all parties concerned. The participants described frustration at seeing the waiting list grow but being unable to help.

"The waiting list is growing. Other departments of our clinic are overburdened with work. Every day several staff members on the emergency ward have no patient-related work. Who says that we are not allowed to work and why? Is it the leadership team or part of it? I feel as if my professional competence is worthless. The leadership team makes everything so unwieldy and complicated. Why doesn’t somebody speak out and ask the senior physician? We are either too compliant or afraid of conflict."

Several factors such as an increased number of patients and tasks, cutbacks or reorganizations cause stress. There are also patients who need accommodation and/or treatment that cannot be provided within the organization and for whom no one wants to pay, thus they may remain on the ward for a long time.

The participants described seriously ill patients who did not receive treatment, thus the ward became *a terminus for the patient*. Some wards had limited resources. It was intended that these patients should be transferred to other wards with more resources or receive treatment from an external expert, but the waiting list was frequently long and there was often a shortage of beds.

"Two suicide candidates. One anorexic and one refugee with traumatic experiences of a really severe kind. There are no possibilities for crisis treatment because of the long waiting list. We will have to start treatment although the patients are not in the least motivated, they just want to die."

It was not just resources for advanced care that were missing, but also for social activities. Some patients had no planned activities or a care plan. Although on many occasions no activities were organized for the patients, except watching TV, the team members insisted on them getting up in the morning and having breakfast at a fixed time. A ward received an asylum seeker boy who did not need any treatment, but became a patient because his mother was admitted to an adult psychiatric ward.

In certain situations the team members experienced *feelings of helplessness,* as they were unable to act in order to improve the situation. They sensed the refugee families’ feeling of hopelessness. Despite having ‘done everything’, they could see no improvement. It seemed pointless to treat the children of asylum seeker families, as they needed a residence permit more than anything else. Sometimes team members found it difficult to know how to deal with their own negative feelings towards patients who committed serious crimes, were violent or created anxiety for other reasons.

When a team member was offended by a colleague or witnessed a colleague insulting a patient, a parent or another colleague, he/she became distressed, but had no idea how to handle the situation. It appeared that these events and feelings were not communicated to the manager or to others at work, but dealt with privately. One person spoke to a family member, while others wrote that they had felt upset about the situation, taken sick leave or experienced difficulty sleeping.

## Discussion

The results of this study indicate that while many of the problems perceived by staff in other health care areas are present in child and adolescent psychiatry, there are also differences. There seems to be a general problem in that care staff and doctors have different opinions on care measures [6-8]. There were also difficulties related to parents who did not agree with what the staff considered best for the patient [6,8]. Loyalty between colleagues was important and gave rise to some ethical considerations [6,11]. Like in other studies [9,10] coercive measures were seldom considered as ethically problematic. Using coercion against the patient more often tended to cause emotional rather than ethical difficulties. However, in our study, there were also differences compared to the aforementioned studies. Our participants frequently reported that organizational boundaries obstructed work with patients, which led to problems for and ethical considerations by staff. In relation to the work with patients*,* there was very little criticism of routine-centred care*,* which was seen by most of the staff as a prerequisite for a well-functioning ward. However, some staff members criticized the fact that patients were stored away instead of treated because resources for adequate care were lacking.

Professional distance, loyalty to the team and maintenance of routines were stressed but, somewhat surprisingly, not closeness to patients see also [11]. Both patients and staff were expected to adapt to the ward routines. Patients had to get up and have breakfast at a certain time in the morning, despite the fact that there were no organized activities available for them. Some staff members questioned this attitude and dared to be more personal in their contact with the patient, which can be of great importance for the latter’s recovery [5]. However, statements indicating that the staff members actively tried to influence decisions already made or the conduct of their colleagues were rare.

Staff members were concerned about refugee children and their families and took the view that a residence permit was a prerequisite for effective care. Therefore Swedish refugee policy, with its long timeframe for handling cases, was the subject of criticism. It is likely that the staff members were correct; current procedures for dealing with asylum seekers seem to contribute to psychiatric problems in already traumatized refugee families [21]. These or other seriously ill patients in addition to inappropriate behaviour on the part of colleagues could so upset staff members that it was difficult for them to let go of thoughts related to their work when they came home. This is supported by previous research on the management of moral stress, which staff members often perceive to be a personal matter [22]. The immediate manager, who ought to serve as a support at work, was rarely mentioned in the statements. This can be serious, as feelings of powerlessness develop in the absence of response and support from one’s manager [23].

In our analysis of the diaries*,* the concept of ethics and what it means for staff members gave rise to reflections concerning our own pre-understanding as well as the regular use of the concept in the literature. When not providing a definition of the concept, ethics seems to have many meanings for staff in child and adolescent psychiatric in-patient care. One staff member wrote: “No specific situation with an ‘ethical dilemma’ this week”. Others described various events, from encounters with patients to organizational problems and their own feelings, sometimes without specifying in what way an ethical consideration was involved. Another observation is that a large and important part of the staff members’ ethical considerations was not related to patient encounters, but rather concerned organizational problems as well as issues of cooperation with and loyalty towards colleagues. Thus, some staff members seemed to interpret ethics in a narrow sense as an issue that rarely occurs. Others saw ethics in a broad sense where all kinds of problem were deemed ethical considerations. We had anticipated a greater number of dilemmas; statements containing reflections on different alternative actions in patient care situations, but these were quite rare. The material we received mainly highlighted ethical situations, but they contained little information about staff members’ reflections on various courses of action. Even in the case of a classical dilemma presented in one of the quotations, when a staff member had to tube-feed a patient, the choice between autonomy versus saving a life was not considered an ethical consideration. Our interpretation is that staff members may have lacked a language to express their reflections in ethical terms.

The diary method provided an effective opportunity to collect rich and comprehensive material. However, despite the wealth of material, most of the statements were brief and thereby had considerable limitations in terms of content. As the clinics were small, the informants were guaranteed anonymity, thus we lack relevant additional information about them. Further research in this area should therefore combine the diary method with interviews or observations in order to obtain a more complete understanding of the ethical considerations made by staff in everyday psychiatric practice.

The diaries were useful in our work with ethics and proved an excellent way of making staff members and supervisors interested in ethical questions, as the diaries permitted them to adequately describe their reality. Such reflection provided a good opportunity for staff members to discuss alternative ways of acting and may therefore be a means of changing current approaches towards patients as well as bringing about organizational changes [24]. The unit managers were also happy with these discussions and found the diaries an excellent way of obtaining a picture of the “ethical landscape” used by staff members to guide their professional actions. Despite its scientific shortcomings, the method employed in this study was found to be a useful tool that could enhance the ethical standard of care, which accords with the informants’ perceptions.

## Conclusions

In summary, the result of the study reveals that the ethical issues described by child and adolescent psychiatric in-patient care staff were multifaceted and often related to loyalty and organizational problems that could have considerable influence on the relationship with the patient and the care provided. Organizational problems as well as violations and patients who are seriously ill or find themselves in precarious situations, e.g. refugee families, risk creating a feeling of powerlessness in staff*,* which is apparently difficult to handle*,* especially when there is a lack of support from the manager. As child and adolescent psychiatric care staff appeared to lack a language of ethics, they probably need both an education in ethics and a forum for discussing ethical issues.

## Competing interests

The authors declare that they have no competing interests.

## Authors’ contributions

IE came up with the research idea, designed the study and was responsible for the data collection. The diaries were thematized by VP and KE, supported by IE. VP drafted the manuscript. All authors have contributed to, read and approved the manuscript.

## Supplementary Material

Additional file 1 Complete instructions to participants. Click here for file
