# Supplementary material for: The ethical landscape of professional care in everyday practice as perceived by staff: A qualitative content analysis of ethical diaries written by staff in child and adolescent psychiatric in-patient care
Source: Child Adolesc Psychiatry Ment Health. 2012 Jul 9;6:18. doi: 10.1186/1753-2000-6-18 (PMC3391975; doi:10.1186/1753-2000-6-18)
Supplement: Additional file 1 — Complete instructions to participants. [file 1753-2000-6-18-S1.doc]

# The complete instructions

**Ethical diary**

With this ethical diary we want to try to capture the situations and experiences that you somehow perceived as ethical considerations at work. This can apply to principal issues with an ethical dimension as well as real-world situations in everyday care.

You are free to express yourself as you want. Describe the situations you have experienced include ethical considerations in any way and the thoughts that this raised within you. Please be detailed in your description of the situation as well as about your thoughts.

The diary should be kept during one working week (week xx). Most preferred is that you write down your experiences every day after the end of day. Write on the attached papers and submit the diary in the drawer that will be available in at the ward. The handing in of the dairy should be done as soon as possible after the end of the week. Deadline for handling in the diary is .........

Your participation is anonymous!
